# Emerging and distinct video head impulse test responses in elderly with vestibular symptoms

**DOI:** 10.1016/j.bjorl.2021.02.011

**Published:** 2021-03-20

**Authors:** Muhammed Ayas, Ahmad AlAmadi

**Affiliations:** a University Hospital Sharjah, Audiology Unit, Sharjah, United Arab Emirates; b University of Sharjah, College of Medicine, Sharjah, United Arab Emirates; c Advanced Hearing and Balance Center, Dubai, United Arab Emirates

**Keywords:** Video head impulse test, Dizziness, Elderly, Vestibulo ocular reflex

## Abstract

**Introduction:**

Dizziness has been reported to be the most common symptom in elderly population. Video head impulse test, VHIT, allows clinicians to assess the vestibular function in elderly individuals, during their initial stages of vestibular symptoms. Inferences from VHIT responses were traditionally low vestibulo-ocular reflex gain or a normal vestibulo-ocular reflex gain. However, the possibility of a third and new variant of the vestibulo-ocular reflex gain has not been clinically explored yet.

**Objectives:**

To determine and report distinct patterns of vestibulo-ocular reflex gain using VHIT in elderly individuals with vestibular symptoms.

**Methods:**

Retrospective cross-sectional study was done on a group of elderly patients who were above 70 years of age. These individuals were subjected to VHIT during their symptomatic phase. A vestibulo-ocular reflex gain value between 0.80–01.20 (Horizontal plane) was considered normal. The gain above and below this cutoff range was considered abnormal.

**Results:**

39 elderly patients (15 males and 24 females) whose mean age range was 74.71 years were evaluated for the VHIT response. Vestibulo-ocular reflex gain obtained was categorized into three distinct patterns: (i) normal vestibulo-ocular reflex gain, (ii) reduced vestibulo- ocular reflex gain and (iii) increased vestibulo-ocular reflex gain. The mean vestibulo- ocular reflex gain for both left and right horizontal canals varied significantly between the three groups (*p* < 0.05). No significant effect of age and vestibulo-ocular reflex gain was noted, though vestibulo-ocular reflex gain was higher in 80 years and above age (*p* > 0.05).

**Conclusion:**

Elderly individuals with dizziness may show varying responses with vestibulo-ocular reflex gain during the symptomatic period. The third type of hyperactive vestibule-ocular reflex responses that emerged from the current study were potential indicators of fluid dynamic changes in the inner ear. These responses need to be explored further as it relates to new clinical markers for both peripheral and central vestibular disorders.

## Highlights

• Video head impulse test in elderly individuals with dizziness.
• Third and new variant of Vestibulo Ocular Reflex gain.
• Higher Vestibulo Ocular Reflex gain in elderly patients.
• Fluid dynamic changes in inner ear may alter Vestibulo ocular reflex gain.

## Introduction

Dizziness was reported to be the most common symptom in elderly population.^1^ Individuals above 60 years are at a high risk of fall and injury due to dizziness.^2^, ^3^, ^4^ It has been reported that more than 50% of all accidental deaths were related to falls in elderly.^5^ The prevalence of vestibular disorders in United States is 85% in the population within the age range of 80 years and above.^6^ Moreover, approximately more than one percent of healthcare costs was spent on fall-related direct treatment in Europe.^7^

Progressive changes in the balance function in elderly are complex and multifactorial in nature. The complex interplay between vestibular system, visual system and proprioception is key for the normal balance function.^8^, ^9^, ^10^ The most potent changes occurred in vestibular function, either in peripheral or in the central function.^11^, ^12^ Neuronal loss may create an alteration in peripheral vestibular input in aged individuals. Consequently, changes in the processing of impaired sensory input at the central circuits may aggravate the symptoms.^13^, ^14^, ^15^ Generally, these peripheral and central vestibular changes lead to increased unsteadiness and imbalance. These individuals commonly seek treatment options from physicians. Typically, balance dysfunction in the elderly was treated with a rather conservative approach due to its multifactorial nature.^16^, ^17^

One of the key components in vestibular assessment is measuring the vestibulo-ocular reflex (VOR) gain. Although, it can be done subjectively, in recent times clinics around the world use more objective tools for measuring VOR gain using video head impulse test (VHIT). It measures the VOR gain in the horizontal semicircular canals (HSC) of motion as well as the posterior and anterior semicircular canals.^18^ Since it was first reported by Halmagyi and Curthoys in 1988,^19^ the head impulse test has evolved over the time. VHIT has by far gained its importance in clinical vestibular evaluation in comparison to other recent technological advances in the field.

The output from the VHIT extrapolates the peripheral vestibular response and its function either as normal VOR gain or abnormally reduced VOR gain. The data published from various clinics around world highlighted the superiority of VHIT in routine vestibular assessment and in identification of vestibular weakness through reduced VOR gain.^18^, ^20^, ^21^, ^22^, ^23^ Studies done on various age groups and the effects on VOR gain measures denoted reduced VOR gain with increased age.^18^, ^22^, ^24^

However, there is limited or a clear dearth of information on higher VOR gain in the elderly. Perhaps, this may be an overlooked phenomenon and attributed to equipment-related validation or clinician-related errors. To the best of our knowledge, there are no reported studies or data on varying VHIT gain in symptomatic elderly individuals with walking difficulty due to limited lower limb functions or among those confined to wheelchairs. It is extremely significant for the clinical community to analyze how the symptomatic elderly individuals respond to VHIT measures. Thus, the aim of the current study was to examine and report emerging data on distinct VOR gain measures obtained from VHIT in symptomatic elderly individuals.

## Methods

Retrospective cross sectional study design was employed for the current study. The study was carried out in an Audiology unit at a tertiary care hospital setting from December 2019 to August 2020.The study samples were successive elderly patients with dizziness who visited the unit during the study period. They were above 70 years of age and included both genders. All the subjects signed a consent form before the procedures. This was in accordance with the Code of Ethics of the World Medical Association (Declaration of Helsinki). Ethical approval was obtained for the current study from the Institutional Ethics and Research Committee (IREC) (Ref. nº UHS-HERC-042-15092020).

The recruited individuals were difficult to test patients who were chronically ill with multiple neuro-orthopedic conditions and were unable to stand or move without support prior to the vestibular symptoms. Those with poor vision and difficultly in neck movements were excluded from the study. All the patients underwent detailed case history taking and vestibular tests including oculomotor tests, positioning tests, pure tone audiometry (PTA) and VHIT. In order to reduce the discomfort, tests such as VHIT and PTA were performed while the patients were seated in the wheelchair. Subsequently, they were shifted to the examination chair/bed for the rest of the clinical evaluation.

### VHIT recording procedure

VHIT was administered to all the patients using ICS Otometrics Impulse (Schaumberg, IL) device. VHIT was done while the patients were seated on the wheelchair. All of them had adequate range of neck movement without any pain or restrictions. A high-speed (250 Hz) video camera, with an embedded accelerometer and a gyro meter in the video goggles were used. It recorded the eye movement (right eye) with respect to the head movement. The visual target at the eye level was located on the wall at a distance of 1 m. The video goggle was fitted tightly in order to reduce goggle slippage. In instances where the head band was loose, a piece of foam was kept behind the head to tighten the google.

Calibration (eye position) was done with the patients by asking them to view a laser dot through the goggle on the wall. They were instructed to follow the laser dots, which were presented arbitrarily. If there were any difficulties or calibration errors, the clinician repeated the calibration before proceeding with the recording. Patients were instructed not to touch or move the goggle after calibration. They were further instructed to maintain their gaze on the visual target; keep their neck muscle relaxed and keep their eyes wide open with minimal blinking, if possible.

The examiner stood behind each patient and rotated their head in the horizontal plane in two directions (left and right) resulting in the stimulation of the left and right HSC. Head movement direction was randomized in order for the patients not to anticipate the head direction. Each subject was put through two sets of recordings for the VHIT responses, in order to efficiently rule out any clinician- related, or equipment- related errors. They underwent a minimum of 20 head impulses in each direction. However, during the recording if a satisfactory impulse was obtained before reaching the 20 head impulses, the recordings were terminated.

### VHIT (VOR) outcome parameters

The parameters of interest were the VOR gain after the head movement. The VOR gain for leftward and rightward head impulses were measured. VOR gain between 0.80–01.20 was considered normal. VOR gain value below 0.80 was grouped as low VOR gain category. Additionally, the gain value of 01.20 or higher was categorized high VOR gain. Both high and low responses were considered abnormal.

### Statistical analysis

The obtained data was entered and analyzed using Statistical Package for the Social Sciences (SPSS) version 22. The categorical data was descriptively analyzed and expressed in percentages. In order to measure the continuous variables (age and VOR gain for left HSC and right HSC), measurements of central tendency (mean and median), dispersion (standard deviation), and position (maximum and minimum) were done. The study cohort was further divided into two age groups and comparisons were made for the age between 70–79 years and 80-years and above. Pearson’s Chi-Square test was used to compare the proportions and unpaired *t*-test was used to compare the VOR gain between the two age groups. Test of normality was done using Shapiro Wilks test. One-way ANOVA was used to compare the VHIT score between the three categories. Pearson’s correlation coefficient was computed to assess the relationship between age and the VOR gain for both the sides; *p*-value of less than 0.05 was taken to define the level of significance.

## Results

The study cohort consisted of 39 participants with a high prevalence of females 24 (61.50%) and 15 (38.50%) males. The age ranged between 70–90 years with a mean age of 74.20 years and standard deviation of 5.01. The participants were further categorized into two age groups: 70–79 years consisting of 31 (79.50%) and 08 (20.50%) in the 80 years and above category.

The clinical features exhibited by the subjects in the two age groups were summarized in Table 1. It can be seen that no participants in the study group had spontaneous nystagmus. In terms of tinnitus perception, 46.16% had left ear tinnitus and 30.80% had right ear tinnitus. Chi-Square test revealed no significant relationship between the 2 age groups for both left (*x*^2^[2, n = 39] = 0.30, *p* > 0.05) and right tinnitus (*x*^2^[2, n = 39] = 0.16, *p* > 0.05).

**Table 1:** Clinical features exhibited by the study cohort.

	Clinical features	Laterality	Age (years)		*X^2^*	*p*-value
			70–79	80 and above		
1	Spontaneous Nystagmus	–	–	–	–	–
2	Tinnitus	Left	15 (38.46%)	03 (07.70%)	0.303	0.58
		Right	10 (25.64%)	02 (05.12%)	0.157	0.70
		Absent	06 (15.38%)	03 (07.70%)	–	–
3	Aural Fullness	Left	07 (17.95%)	04 (10.26%)	2.361	0.12
		Right	04 (10.26%)	02 (05.13%)	0.715	0.40
		Absent	20 (51.28%)	02 (05.13%)	–	–
4	Hearing loss	Left	13 (33.33%)	04 (10.26%)	2.226	0.70
		Right	10 (25.64%)	04 (10.26%)	4.810	0.31
		Absent	08 (20.51%)	0	–	–

*p*-values were calculated using Chi-Square tests (*X^2^*).

Significant at *p* ≤ 0.05 level.

Aural fullness was reported in 28.20% in the left ear and 15.38% in the right ear. There was no statistically significant correlation between the age and the effect on the left (*x*^2^[2, n = 39] = 2.40, *p* > 0.05) and the right ear (*x*^2^[2, n = 39] = 0.71, *p* > 0.05) aural fullness.

The hearing sensitivity varied significantly within the study group with degree of sensorineural hearing loss ranging from mild to severe hearing loss with a combined percentage of 43.58% in left ear and 35.90% in right ear respectively (Table 1). There was no effect of age on laterality of hearing sensitivity (left ear (*x*^2^[2, n = 39] = 2.23, *p* > 0.05) and right ear (*x*^2^[2, n = 39] = 4.81, *p* > 0.05).

Analysis of the combined 78 (39 × 2) VHIT responses for left HSC and right HSC VOR gain categories was expressed in Table 2. 14 (35.90%) patients had left VHIT (mean = 0.90; SD = 0.07) and 16 (41.02%) had right VHIT (mean = 0.89; SD = 0.09) within the normal range (VOR gain value of between 0.80–01.20). The reduced VOR gain category (less than 0.80) was reported showing 15 (38.46%) left VHIT (mean = 0.56; SD = 0.16) and 13 (33.33%) right VHIT (mean = 0.50; SD = 0.16).

**Table 2:** Mean and standard deviation (SD) of VOR gain for the left and right HSC.

VOR gain	Left VOR gain (n = 39)		Right VOR gain (n = 39)	
	n (%)	Mean (SD)	n (%)	Mean (SD)
Normal (0.80–01.20)	14 (35.90)	0.90 (0.07)	16 (41.01)	0.89 (0.09)
Low (<0.80)	15 (38.51)	0.56 (0.16)	13 (33.33)	0.50 (0.16)
High (>01.20)	10 (25.60)	1.34 (0.83)	10 (25.60)	1.35 (0.11)

VOR, Vestibulo-ocular reflex; HSC, Horizontal semicircular canals.

Additionally, the increased VOR gain category (VOR gain more than 01.20) revealed, 10 (25.64%) left VHIT (mean = 1.34; SD = 0.08) and 10 (25.64%) right VHIT (mean = 1.35; SD = 0.11). VOR gain in this group was above the cutoff value.

The unpaired *t-*test performed to compare the VOR gain between the two age groups (70–79 years and 80 years above) showed no significant difference between the age groups (*p* > 0.05). However, the VOR gain was higher in the 80 years and above age group (Table 3).

**Table 3:** *t-*test results for the comparison of the VOR gain in two age groups.

	*t*-test for equality of means				
	*t*	*p*	Mean difference	95% Confidence Interval of the difference	
				Lower	Upper
VOR L	−0.575	0.569	−0.07629	−0.34498	0.19240
VOR R	−0.217	0.829	−0.03077	−0.31759	0.25606

VOR, Vestibulo-ocular reflex.

In order to compare the VOR gain with the three defined variance, one-way ANOVA was performed for left and right VOR gain. Prior to conducting the ANOVA, the test of normality was done for continuous data by using Shapiro Wilks test and the data was normally distributed. ANOVA compared the three VOR outcome parameters and found that mean VOR gain was significantly higher in 01.20 cut off value group for both left (*F =* 125.00; *p* = 0.001*)* and right side (*F =* 132.44; *p* = 0.001) and VOR gain was lowest in the less than 0.80 cut off group. The mean VOR gain differed significantly between the groups (*p* < 0.05). To further assess the nature of the differences among the three VOR categories after ANOVA, Bonferroni Multiple comparisons post hoc test was performed. The difference between the lower VOR gain group and higher VOR gain group was statistically significant in both left and right side respectively (*p* < 0.05). Also, a Pearson’s correlation coefficient was computed to assess the relationship between age and the VOR gain for the left (r = 0.14) and right (r = 0.07) side and found weak positive correlation (*p* > 0.05).

## Discussion

The aim of the current study was to examine and report distinct levels of VOR gain responses with VHIT in older symptomatic patients. The results were encouraging, and we believe this will be the first data set report on the varying gain in the elderly cohort. The outcome of the current study revealed that the VHIT responses were varied in its nature. This was in contrast to the traditional responses of either normal or low VOR gain reported in the literature. The responses noted in the current study were either normal VOR gain, low VOR gain or a higher VOR gain.

Patients who reported normal VOR gain with vestibular symptoms were diagnosed to have positional vertigo. This was established through the positioning tests performed during the clinical evaluation. Majority had posterior BPPV (benign paroxysmal positional vertigo) and the rest had lateral canal BPPV. No anterior canal BPPV was reported. This was in agreement with the reported findings in literature that, in cases of BPPV, VHIT is more often reported to be normal with preserved HSC function.^25^ Also, there were findings that in acute vestibular syndrome (AVS), VHIT may be normal in HINTS (head impulse nystagmus test of Skew) test.^26^

Reduced VOR gain was seen in the patients with acute or chronic vertigo. In such patients, the VHIT responses were reported to be below the normal range either unilaterally or bilaterally.^23^ Considering the current group of individuals in the study, the findings may also correlate with the age-related changes in the vestibular system. The reduced gain could be attributed to the continuous degenerative neuronal loss or at the level of Scarpa ganglion.^15^ In a study by Agarwal et al.,^27^ it is reported that individuals who are above 70 years showed a global decline in peripheral vestibular end organ functions, the majority of whom had semicircular canal dysfunction. Temporal bone and pathologic studies have shown that there was significant loss of sensory hair cells in the cristae ampularis compared to the maculae. It was worth noting that, even during the age-related decline, relative sparing of the utricular and saccular function were seen.^28^, ^29^, ^30^ However, such precise interpretation was possible only when these structures were assessed with cervical and ocular vestibular evoked myogenic potentials (VEMP) along with VHIT measures.^27^

Interestingly, we found a third group of VHIT response in the current cohort: higher or increased VOR gain. To our knowledge, the current data from the study would be the first to report such variant VHIT results in elderly population. This group had shown higher VOR gain on repeated trail of VHIT, which excluded any instrument, subject or user related errors. Although it is an uncharted territory, we believe that there could be a multifactorial explanation for the higher VOR gain. Angeli et al.^31^ reported that hyperactive peripheral responses were seen in a cohort of 11 subjects, on whom they studied caloric test and eccentric subjective visual vertical test (SVV). They believed that the hyper responses were due to the canal interactions and utricular hyperfunction. Though this was an interesting finding, substantial evidence was rarely reported regarding the hyperactive VHIT responses.

Another possible explanation for the higher VOR gain responses could relate to the fluid dynamic changes in the inner ear structures. Though it is not uncommon for the individuals in older age group to have endolymphatic hydrops or Meniere’s disease (MD), it would be challenging for the clinicians to reach a conclusive diagnosis, especially due to the decreased postural and gait control and the multiple comorbidities present at old age.^32^ However, in the authors clinical experience in a younger adult population, there was a correlation found between high VOR gain in MD patients. This group of patients were definite MD on whom VHIT gain were extremely high on the affected side. These findings were presented for the journal review at the time of writing the current study. Contradicting reports were available in literature on unaffected VHIT responses for MD subjects.^33^, ^34^

Additionally, we believe that the results of our study could indicate possible central vestibular involvement. Abnormally increased excitatory responses in the central vestibular pathway may cause hyperactive responses. It can stem from the lack of cerebellar inhibition. During the vestibular afferent stimulation, normally fastigial fibers create a negative loop for inhibition. However, if any disruptions, due to lesions in the vestibular nucleus or medial longitudinal fasciculus, it may exhibit different responses.^35^

In summary, the current data exhibits a window to new and emerging trends in VHIT, which were not reported previously. The study results were varied in its nature within the same group. It sheds light into the inherent and more complex nature of the aging vestibular system. Dearting from the traditional VHIT responses, it is important for clinicians to expect higher VHIT responses in symptomatic individuals, particularly in older age groups.

### Limitations of the study

Though the study results offered interesting findings, the current study had its limitations. Data was limited in number and specific grouping was not done at the entry level. Also, only HSC was measured with VHIT. Additional measures such as VEMPs were not done to measure the responses.

### Future directions

The present study has opened a window for different study designs involving VHIT in the future. Depending on the availability, a control group can be added to compare the results. Future studies can also focus more on aligning the VEMPs and VHIT responses in older adults. Additionally, ECOG (electrocochleography) and VHIT can be used to investigate the relationship between higher VOR gain and ECOG responses. The hyperactive responses are of particular interest and need to be explored further.

## Conclusion

The current study was an attempt to report the varied VOR gain obtained from VHIT. The findings shed light into the complex nature of the aging vestibular system. The study revealed a variant of VHIT response with higher VOR gain, in addition to the traditional VHIT responses. The third type of hyperactive VOR responses that emerged from the current study were potential indicators of fluid dynamic changes in the inner ear. These responses need to be explored further by the clinical and research community, as it could relate to new clinical markers for both peripheral and central vestibular disorders. Future studies can focus on improved sampling with wider study groups and explore in detail about the hyperactive VHIT responses.

## Funding

This research did not receive any specific grant from funding agencies in the public, commercial, or not-for-profit sectors.

## Conflicts of interest

The authors declare no conflicts of interest.

## References

[1] Furman J.M., Raz Y., Whitney S.L. (2010). Geriatric vestibulopathy assessment and management. Curr Opin Otolaryngol Head Neck Surg..

[2] Alyono J.C. (2018). Vertigo and dizziness: understanding and managing fall risk. Otolaryngol Clin North Am..

[3] Verghese J., Ambrose A.F., Lipton R.B., Wang C. (2010). Neurological gait abnormalities and risk of falls in older adults. J Neurol..

[4] Davis L.E. (1994). Dizziness in elderly men. J Am Geriatr Soc..

[5] Rauch S.D., Velazquez-villaseñor L.U., Dimitri P.S., Merchant S.N. (2001). Decreasing hair cell counts in aging humans. Ann N Y Acad Sci..

[6] Agrawal Y., Carey J.P., Della Santina C.C., Schubert M.C., Minor L.B. (2009). Disorders of balance and vestibular function in US adults: data from the National Health and Nutrition Examination Survey, 2001-2004. Arch Intern Med..

[7] Jahn K., Kressig R.W., Bridenbaugh S.A., Brandt T., Schniepp R. (2015). Dizziness and unstable gait in old age: etiology, diagnosis and treatment. Dtsch Arztebl Int..

[8] Iwasaki S., Yamasoba T. (2015). Dizziness and imbalance in the elderly: age-related decline in the vestibular system. Aging Dis..

[9] Wiesmeier I.K., Dalin D., Maurer C. (2015). Elderly rely on proprioception rather than on space cues when standing. Front Aging Neurosci..

[10] Illing S., Choy N.L., Nitz J., Nolan M. (2010). Sensory system function and postural stability in men aged 30–80 years. Aging Male..

[11] Zalewski C.K. (2015). Aging of the human vestibular system. Semin Hear..

[12] Anson E., Jeka J. (2016). Perspectives on aging vestibular function. Front Neurol..

[13] Allen D., Ribeiro L., Arshad Q., Seemungal B.M. (2017). Age-related vestibular loss: current understanding and future research directions. Front Neurol..

[14] Merchant S.N., Tsuji K., Wall I.I.I.C., Velázquez-Villaseñor L., Glynn R.J., Rauch S.D. (2000). Temporal bone studies of the human peripheral vestibular system: 1. Normative vestibular hair cell data. Ann Otol Rhinol Laryngol Suppl..

[15] Park J.J., Tang Y., Lopez I., Ishiyama A. (2001). Age-related change in the number of neurons in the human vestibular ganglion. J Comp Neurol..

[16] Stam H., Van Der Wouden J.C., Hugtenburg J.G., Twisk J.W., Van Der Horst H.E., Maarsingh O.R. (2018). Effectiveness of a multifactorial intervention for dizziness in older people in primary care: a cluster randomised controlled trial. PLoS ONE..

[17] Kendall J.C., Hartvigsen J., Azari M.F., French S.D. (2016). Effects of nonpharmacological interventions for dizziness in older people: systematic review. Phys Ther..

[18] McDougall H.G., McGarvie L.A., Halmagyi G.M. (2009). The video head impulse test: diagnostic accuracy in peripheral vestibulopathy. Front Neurol..

[19] Halmagyi G.M., Curthoys I.S. (1988). A clinical sign of canal paresis. Arch Neurol..

[20] Alhabib S.F., Saliba I. (2017). Video head impulse test: a review of the literature. Eur Arch Otorhinolaryngol..

[21] Lee J.Y., Park J.S., Kim M.B. (2019). Clinical characteristics of acute vestibular neuritis according to involvement site. Otol Neurotol..

[22] Janky K.L., Patterson J., Shepard N., Thomas M., Barin K., Creutz T. (2018). Video head impulse test (vHIT): the role of corrective saccades in identifying patients with vestibular loss. Otol Neurotol..

[23] Stevens M.N., Garrison D.B., Kaylie D.M. (2017). What is the potential clinical utility of vHIT when assessing adult patients with dizziness?. Laryngoscope..

[24] Pogson J.M., Taylor R.L., Bradshaw A.P., McGarvie L., D’Souza M., Halmagyi G.M. (2019). The human vestibulo-ocular reflex and saccades: normal subjects and the effect of age. J Neurophysiol..

[25] Çınar Y., Bayram A., Culfa R., Mutlu C. (2018). Analyses with the video head impulse test during the canalith repositioning maneuver in patients with isolated posterior semicircular canal benign paroxysmal positional vertigo. Turk Arch Otorhinolaryngol..

[26] Kattah J.C. (2018). Use of HINTS in the acute vestibular syndrome. An overview. Stroke Vasc Neurol..

[27] Agrawal Y., Zuniga M.G., Davalos-Bichara M., Schubert M.C., Walston J.D., Hughes J. (2012). Decline in semicircular canal and otolith function with age. Otol Neurotol..

[28] Rosenhall U. (1973). Degenerative patterns in the aging human vestibular neuro-epithelia. Acta Otolaryngol..

[29] Igarashi M., Saito R., Mizukoshi K., Alford B.R. (1993). Otoconia in young and elderly persons: a temporal bone study. Acta Otolaryngol Suppl..

[30] Ono K., Keller J., Ramírez O.L., Garrido A.G., Zobeiri O.A., Chang H.H. (2020). Retinoic acid degradation shapes zonal development of vestibular organs and sensitivity to transient linear accelerations. Nat Commun..

[31] Angeli S.I., Snapp H., Velandia S., Morgenstein K. (2015). Utricular paresis and semicircular canal hyperactivity: a distinct otolith syndrome. Acta Otolaryngol..

[32] Teggi R., Meli A., Trimarchi M., LiraLuce F., Bussi M. (2012). Does Ménière’s disease in the elderly present some peculiar features?. J Aging Res..

[33] Hannigan I.P., Welgampola M.S., Watson S.R. (2019). Dissociation of caloric and head impulse tests: a marker of Meniere’s disease. J Neurol..

[34] Martinez-Lopez M., Manrique-Huarte R., Perez-Fernandez N. (2015). A puzzle of vestibular physiology in a Meniere’s disease acute attack. Case Rep Otolaryngol..

[35] Lee S.H., Kim S.H., Kim J.M., Tarnutzer A.A. (2018). Vestibular dysfunction in Wernicke’s encephalopathy: predominant impairment of the horizontal semicircular canals. Front Neurol..

